# Aphid-ResNetSwin: An Image Recognition Method with Improved Attention Mechanism for Graded Identification of *Myzus persicae*

**DOI:** 10.3390/insects17030305

**Published:** 2026-03-11

**Authors:** Jinzhou Luo, Jiazhao Sun, Xiaoli Hao, Heng Liu, Fajin Lv, Wei Ding

**Affiliations:** 1 College of Plant Protection, Southwest University, Chongqing 400715, China; luojinzhou@email.swu.edu.cn (J.L.);; 2 School of Sociology, Guizhou Minzu University, Guiyang 550025, China

**Keywords:** *Myzus persicae*, infestation severity, Aphid-ResNetSwin, GCSA, dual-branch hybrid neural network

## Abstract

*Myzus persicae* is the most devastating piercing-sucking pest threatening tobacco production. Precise quantification of infestation severity is a prerequisite for precision pest management, making the integration of visual image analysis highly essential for efficient management. Current computer vision models in modern agriculture are primarily designed for classifying various pest species, and there is a lack of image-driven analytical tools for assessing the severity of damage inflicted by a single target pest. To supplement existing analytical tools and enable the graded recognition of tobacco aphid (*M. persicae*) infestation levels, we propose the Aphid-ResNetSwin model. This model is constructed by integrating a Global Channel-Spatial Attention module (a specialized attention mechanism) into the well-established InceptionResNetV2 architecture. Performance evaluation results demonstrated that the Aphid-ResNetSwin model achieved a graded recognition accuracy of 89.11%. Compared with widely adopted mainstream baseline models in computer vision, such as MobileNetV3, SwinTransformer, and InceptionResNetV2, our proposed model exhibited superior performance in recognition accuracy. Furthermore, the classification accuracy of our model for *M. persicae* infestation across all severity levels was significantly higher than that of manual identification, with the exception of healthy leaves. Collectively, our findings indicate that the Aphid-ResNetSwin model provides a robust tool for the graded recognition of *M. persicae* infestation, offering valuable model-based support for the precision control of aphids in tobacco fields.

## Simple Summary

Infestation of crops by the *Myzus persicae* results in yield loss and quality deterioration of agricultural products. Accurately identifying *M. persicae* helps to develop prevention and control strategies in advance, thereby reducing related yield and quality losses. Traditional image classification methods exhibit significant limitations in terms of accuracy and robustness under complex field conditions. To address these challenges, this study proposes a novel image recognition model, Aphid-ResNetSwin, for the graded identification of tobacco aphids. This network employs a novel dual-branch hybrid neural network architecture based on Inception-ResNet-V2 and Swin Transformer, in which the Global Convolutional Spatial Attention (GCSA) module is integrated into each branch to enhance feature attention extraction. Such a design effectively improves the capability of local feature learning and recognition accuracy, thereby significantly boosting the overall recognition performance.

## 1. Introduction

Precision pest identification are critical for sustainable agricultural production, serving as a foundational component for implementing Integrated Pest Management (IPM) strategies and driving advancements in precision agriculture [1,2]. By enabling accurate monitoring and timely early warning, pest predictive models facilitate the optimization of control tactics, leading to reduced pesticide application, improved agricultural product quality and yield, and the preservation of ecological balance. In conventional pest research, pest monitoring and surveys are primarily conducted on-site by professional technicians, thus yielding relatively accurate and reliable datasets [3]. However, this approach is time-consuming, labor-intensive, costly, and potentially harmful to the environment, rendering it incompatible with the requirements of large-scale applications. However, conventional pest identification approaches remain constrained by inherent limitations, including reliance on empirical knowledge, insufficient real-time adaptability, high technical complexity, and elevated operational costs [4,5]. These constraints significantly hinder the practical implementation of traditional methods, necessitating the development of digital monitoring models to address the emerging needs of modern pest management.

Tobacco, as a multi-attribute cash crop, holds significant economic value and serves as an economic pillar in specific regions [6,7]. Therefore, improving the quality and yield of tobacco is a key focus of agricultural poverty alleviation [8]. However, tobacco cultivation is frequently compromised by pest infestations, leading to stunted growth or physiological disorders. The *Myzus persicae* stands out as a predominant pest, with aphids being specialized phloem-feeding herbivores in vascular plants [9]. The *M. persicae* not only causes physical damage to tobacco leaves and stems but also acts as vectors for viral transmission, exacerbating plant disease incidence [10,11]. Effective control of *M. persicae* populations is therefore critical for maintaining tobacco leaf productivity. The identification of different levels of aphids on tobacco leaves can accurately identify the current damage of tobacco leaves and carry out precise control. This strategy enhances pest management efficiency and strengthens the agricultural sector’s resilience to biotic risks.

In recent years, image recognition technology has been widely applied in crop pest identification and achieved promising results [12,13,14]. Among various deep learning architectures, InceptionResNetV2 integrates the multi-scale feature extraction mechanism of the Inception series with the residual connection technology of ResNet. This integration not only maintains strong transfer learning and generalization capabilities but also enables faster convergence than traditional architectures, attributed to its Inception structure with residual connections [15,16]. Some researchers adopted an improved YOLOv5 model to identify the number of individual aphids, achieving a detection accuracy of over 99% for *M. persicae* in greenhouse environments [17]. Some researchers adopted a modified YOLOv5 model combined with data augmentation for the individual detection of Aphis gossypii, achieving a mean average precision (mAP) of 95.7%, which outperformed the comparative SSD and Faster R-CNN models [18].

However, the differentiation of *M. persicae* at different infestation levels relies on subtle density differences, and the deep features extracted by InceptionResNetV2 may lose such small-target details [17]. To address this issue, we introduced the channel attention mechanism of Global Channel-Spatial Attention (GCSA) to adaptively enhance key feature channels, thereby improving the model’s discrimination accuracy for similar categories [19]. The acquisition of *M. persicae* infestation images faces three major challenges: seasonal window limitations (aphids are highly prevalent only during the vigorous growth stage of tobacco), high annotation costs (requiring experts to count aphids one by one), and extreme class imbalance (samples with severe infestation ac-count for less than 5%). Traditional data augmentation techniques (e.g., rotation, flip-ping, and brightness adjustment) can improve the robustness of InceptionResNetV2, but their ability to preserve the vision of aphid aggregation patterns is limited [20]. Therefore, we further augmented the *M. persicae* image dataset based on generative adversarial networks (GANs) [21]. This method has been extensively adopted in the image augmentation techniques for crop pest monitoring [22,23,24]. To address the aforementioned problems, we propose a grading recognition model for the severity of *M. persicae* damage, which adopts a dual-branch hybrid neural network architecture based on Inception-ResNet-V2 and Swin Transformer. Meanwhile, the Global Convolutional Spatial Attention (GCSA) module is integrated into the branches to enhance the extraction of attention features.

In this study, tobacco was adopted as a research case, and the primary contributions are summarized as follows:

1. In this study, a dual-branch hybrid neural network architecture is designed, which fuses the local feature extraction capability of InceptionResNet-V2 with the global modeling capability of Swin Transformer.
2. The recognition performance of the model is enhanced by incorporating a global channel-spatial attention module (GCSA) at the end of each branch.
3. The proposed Aphid-ResNetSwin model achieves accurate identification of *M. persicae* infestation severity and outperforms human-based recognition in terms of accuracy.

## 2. Materials and Methods

### 2.1. Field Collection

Image acquisition was conducted in tobacco-growing farmlands located in the mountainous areas of southeastern Guizhou Province, a region characterized predominantly by hilly and mountainous terrain. Where tobacco is conventionally field-grown from late April to early August, with the peak population dynamics of *M. persicae* occurring primarily from April to June. The tobacco cultivar “Yunyan87” was selected, planted at a density of 1100 plants per 667 m^2^. Developmental stages of tobacco throughout the growing season are summarized in Table 1. *M. persicae* images were collected using a random sampling approach, with image samples acquired under visible light conditions including photographs against simple and complex backgrounds. Images were captured without using a macro lens for magnification; instead, focus was carefully adjusted to align with tobacco leaves. Aphid-induced damage to tobacco primarily occurs from the rosette stage to the rapid growth stage, defined as the 60-day period following transplanting of seedlings from the nursery to the field. Image acquisition was conducted within this timeframe. Post-collection, images were classified into three damage severity grades based on aphid population density, following the criteria outlined in “The response of the red morph of the tobacco aphid, *M. persicae*, to insecticides applied under laboratory and field conditions.” [25]. Images were captured under diverse outdoor lighting conditions, including clear/bright, cloudy, and partially cloudy skies. The equipment parameters used for model training are listed in Table 2.

### 2.2. Image Preprocessing

Image preprocessing can help improve the generalization ability of classification system by adjusting the distribution of training and test data [26]. First, all images were resized to 299 × 299 pixels to ensure consistent input dimensions for the model. Simple data augmentation techniques—including random rotation, color jittering, and grayscale adjustment—were applied to improve model generalization. Processed images were converted to tensor format, with pixel values normalized to the [0,1] range and reshaped from HWC (height–width–channel) to CHW (channel–height–width) format. Each channel was then standardized through normalization. To further enhance data diversity and improve model generalization and robustness, this study integrated a Generative Adversarial Network (GAN) [27]. The GAN was optimized to minimize the Jensen–Shannon (JS) divergence between generated and real data distributions, with the generator taking Gaussian noise as input. The loss function optimization process can be expressed as:

(1) $$L\left(G,D\right)=\underset{G}{\min}\;\underset{D}{\max}\left\{\underset{x⟶P_{r}\left(z\right)}{E}\left[logD\left(x\right)\right]+\underset{z⟶P_{g}\left(z\right)}{E}\left[log\left(1-D\left(G\left(Z\right)\right)\right)\right]\right\}$$

In the formula, E[ ] denotes the expectation function; G[ ] represents the generator function; D[ ] signifies the discriminator function; Pr[ ] indicates the distribution of target data x; Pg[ ] denotes the distribution of noise data; Z is the input noise data vector; and x is the real data sample.

The generator loss function can be expressed as the following formula:

(2) $$L_{G}=-\underset{x⟶P_{g}\left(z\right)}{E}\left[D\left(G\left(z\right)\right)\right]\;$$

The loss function of the discriminator can be expressed as follows:

(3) $$L_{D}=-\left(\underset{x⟶P_{r}\left(x\right)}{E}\left[logD\left(x\right)\right]+\underset{\hat{x}⟶P_{g}\left(\hat{x}\right)}{E}\left[log\left(1-D\left(\hat{x}\right)\right)\right]\right)$$

### 2.3. Image Recognition Model Architecture

The InceptionResNetV2 network was employed as the feature extraction framework. This structure integrates Inception modules and ResNet residual connections, leveraging ResNet’s design to train deeper layers without overfitting. The Inception modules utilize 1 × 1 convolutions and replace traditional pooling layers with global average pooling, reducing computational complexity and model parameters [28,29]. However, in view of the multi-scale characteristics of the damage symptoms caused by *M. persicae*, InceptionResNetV2 exhibits a slight deficiency in global feature extraction capability. To address this issue, we introduce Swin Transformer for global attention extraction [30]. In this study, a dual-branch hybrid neural network architecture (Aphid-ResNet-Swin) is proposed, which achieves accurate damage grade recognition by fusing the local feature extraction capability of convolutional neural networks with the global modeling capability of vision transformers. The model network proposed by this research institute consists of three core components:

(4) $$M=(E_{cnn},E_{trans},F_{fusion})$$

$E_{cnn}$, the convolutional branch based on InceptionResNetV2, is responsible for extracting local fine-grained features;

$E_{trans}$, the Transformer branch based on Swin Transformer, undertakes the modeling of global contextual relationships;

$F_{fusion}$, the feature fusion and classification head, enables the collaborative decision-making of dual-branch features.

Preprocessed input images (resized to 224 × 224 and normalized) were fed into the dual-branch encoder in parallel. The upper branch adopted Inception-ResNet-V2, which extracted 1536-dimensional local features through a Stem module (Conv + Pool × 7, output: 71 × 71 × 192), a stack of Inception-ResNet Blocks (Block-A × 10, Block-B × 20, Block-C × 10, incorporating multi-scale convolutions and residual connections), and global average pooling (GAP). The lower branch employed Swin Transformer, which generated 768-dimensional global features via Patch Embedding (4 × 4 patch partitioning, output: 56 × 56 × 96), four stacked shifted window multi-head self-attention modules (Stage 1–4, with a hierarchical design), as well as Layer Normalization (Layer Norm) and GAP. After the dual-branch features were adaptively enhanced by the GCSA module (consisting of channel attention and spatial attention) respectively, they were concatenated in the adaptive feature fusion layer (1536 + 768 = 2304 dimensions). Through attention weighting and feature interaction in sequence, the fused features were finally input into the MLP classification head (FC(2304 → 1024) → BN + ReLU → Dropout(0.4) → FC(1024 → 512) → FC(512 → 4)), and the probability distribution of four classes was output via the Softmax function.

**Figure 1:**
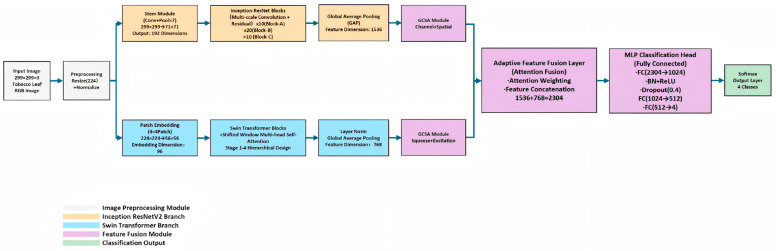
Schematic diagram of the fusion architecture recognition model involved in this study.

To enhance the discriminability of features, a global channel-spatial attention module (GCSA) is introduced at the end of each branch. This module generates complementary attention weights through a parallel channel attention branch (global average pooling-multilayer perceptron-Sigmoid activation) and a spatial attention branch, and realizes feature recalibration via residual connections. The overall architecture is illustrated in Figure 1.

A hierarchical and differentiated weight initialization scheme was adopted in this study. For the Inception-ResNet-V2 branch, the pre-trained weights on ImageNet-1K were loaded to inherit the robust capability of local feature extraction; for the Swin Transformer branch, the pre-trained weights on ImageNet-22K were adopted to capture more extensive global contextual dependencies. The convolutional layers of GCSA were initialized with the Kaiming normal initialization to match the variance characteristics of the ReLU activation function, while the fully connected layers of the adaptive feature fusion layer and the MLP classification head were initialized with the Xavier uniform initialization to ensure the scale consistency of forward propagation and backward gradients. In addition, an early stopping mechanism was set up, which triggers a stopping check when the validation loss does not decrease for 10 consecutive epochs.

This mechanism enables the model to focus on critical regions of the image, enhancing feature representation and distinguishing aphids of varying morphologies from tobacco leaf backgrounds. Add Rectified Linear Unit (ReLU) as an activation function during use to solve the gradient vanishing problem and speed up the training process. The proposed model was benchmarked against conventional classification architectures—Inception ResNetV2, EfficienNetV2, and MobileNet V3—to demonstrate its superiority in tobacco aphid recognition. Performance comparisons were conducted across key metrics, including classification accuracy, F1-score, inference time, and model size, under identical experimental conditions. This comprehensive evaluation aimed to validate the model’s advancement in addressing the specific challenges of small-object detection in complex agricultural environments.

### 2.4. Optimization of Aphid Recognition Model with GCSA Module

**Figure 2:**
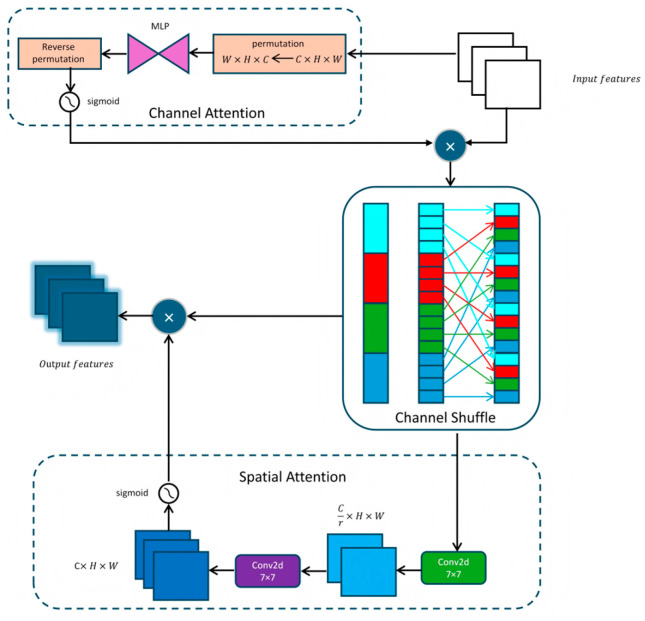
GCSA mechanism structure.

Considering that the fusion strategy may treat noisy features and discriminative features equally, which is prone to induce feature cancellation and gradient conflict and restrict the model’s capability to capture critical visual patterns, a global channel-spatial attention mechanism (GCSA) is proposed in this study. Specifically, parallel channel and spatial attention branches are introduced at the end of each branch, and adaptive feature recalibration is achieved via residual connections, thus accomplishing feature purification and semantic alignment prior to feature fusion (Figure 2) [31].

In the channel attention submodule, the input feature map first undergoes dimension permutation, transforming from the C × H × W format to W × H × C. Subsequently, a two-layer multilayer perceptron (MLP) is employed to capture the interdependencies among channels. The first MLP layer reduces the number of channels to one-fourth of the original dimension, followed by the introduction of non-linearity via the ReLU activation function; the second MLP layer then restores the channel dimension to its original size. Finally, an inverse permutation is performed to revert the feature map to the C × H × W format, and a channel attention map is generated through the Sigmoid activation function. The enhanced feature map is obtained by conducting an element-wise multiplication between the input feature map and the channel attention:

(5) $$F_{channel}=\sigma (MLP(Permute(F_{input})))⊙F_{input}$$

$F_{channel}$ is the enhanced feature map, σ is the Sigmoid function, ⊙ representing element wise multiplication, and $F_{input}$ is the original input feature map.

To further mix and share information, apply channel shuffling operations. The enhanced feature maps are divided into (4) groups, each containing (C/4) channels. Transpose the grouped feature maps to shuffle the channel order within each group. Subsequently, the scrambled feature map is restored to its original shape (C × H × W). This approach can better mix feature information and enhance feature expression ability.

(6) $$F_{shuffle}=ChannelShuffle(F_{channel})$$

$F_{shuffle}$ is the mixed feature map, and $F_{channel}$ is the number of channels in the input feature map.

In the spatial attention submodule, the input feature map is passed through a 7 × 7 convolutional layer, which reduces the number of channels to one-fourteenth of the original dimension. It then undergoes non-linear transformation via batch normalization and the ReLU activation function. Subsequently, a second 7 × 7 convolutional layer restores the channel dimension to the original value C, followed by another batch normalization layer. Finally, a spatial attention map is generated through the Sigmoid activation function. The shuffled feature map and the spatial attention map are subjected to element-wise multiplication, yielding the final output feature map.

(7) Fspatial=σ(Conv(BN(ReLU(Conv(Fshuffle)))))⊙Fshuffle

Fspatial is the feature map after spatial attention.

The final output feature map contains enhanced features after channel attention, channel shuffling, and spatial attention.

### 2.5. Model Validation

In the tobacco aphid image recognition model, the proposed model was compared with the InceptionResNetV2 baseline model, and five-fold cross-validation was adopted to demonstrate the performance of the proposed model. For model performance evaluation, seven metrics were employed to identify the optimal model for tobacco aphid infestation grading, including the average training loss, average test loss, average training accuracy, average test accuracy, precision, recall, and F1-score. In the model training process, the dataset was split into an 80% training set and a 20% validation set in this study.

*P* = *TP*/(*TP* + *FP*) (8)

*R* = *TP*/(*TP* + *FN*) (9)

*F*1 = 2*PR*/(*P* + *R*) (10)

Lower loss values and higher accuracy rates are generally indicative of superior model performance. Each model was trained for 100 iterations, with iteration curves plotted to assess stability. Meanwhile, the model prioritizes true positive predictions, enabling a more nuanced understanding of its performance—particularly when targeting *M. persicae* across different infestation severity levels. Considering model portability during deployment, model size and inference time were adopted as application-specific evaluation metrics. A graphical user interface (GUI) application for image recognition was developed using Python 3.10 Tkinter library to test model recognition accuracy. Specifically designed to evaluate tobacco aphid *M. persicae* damage identification by image recognition models, the interface facilitates easy upload, display, and prediction of pest-infested images. The practical application value of the proposed model was validated by comparing its performance against the accuracy of manual identification. To verify the generalizability and robustness of the proposed Aphid-ResNetSwin model, cross-dataset validation was conducted using a tobacco whitefly (*Bemisia tabaci*) dataset, which is a distinct yet agriculturally important insect pest on tobacco crops. The cross-dataset validation aimed to evaluate whether the model could maintain satisfactory performance when applied to pest recognition tasks beyond the original tobacco aphid training dataset, thereby confirming its potential for wide-ranging practical applications in tobacco pest monitoring.

### 2.6. Model Testing Statistical Analysis

To empirically validate the effectiveness of the proposed model, this study compared the model-based identification with manual identification performance. A total of 500 randomly selected image sets were subjected to grouped identification experiments. For the first group, the proposed model was applied to identify the 500 image sets, and the recognition accuracy was statistically analyzed across four hierarchical levels (healthy, mild damage, moderate damage, and severe damage), with the accuracy of each level calculated independently. For the second group, manual identification was conducted on the same image sets. A stratified sampling strategy was adopted to construct a manual identification panel consisting of 10 participants, including 6 agricultural technicians with more than 3 years of practical experience (representing core judgment capacity) and 4 agricultural growers (representing supplementary labor force), which was designed to simulate the real-world conditions of field production. The panel performed hierarchical identification on the 500 images and the recognition accuracy of each level was quantified as well. Meanwhile, the inter-rater reliability was employed to evaluate the consistency of manual identification results.

## 3. Results

### 3.1. The Effect of Image Augmentation

**Figure 3:**
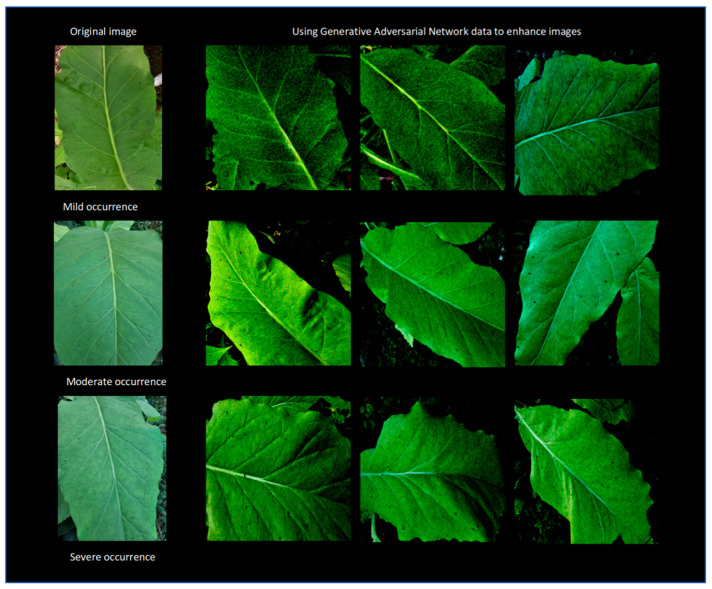
Schematic of image classification for different levels of tobacco *M. persicae* damage and the effect after data augmentation.

A total of 1760 images were collected, including 419 healthy tobacco leaf images (without *M. persicae* attachment), 429 images of low-density *M. persicae* infestation, 516 images of moderate *M. persicae* population, and 396 images of severe *M. persicae* damage. All images were acquired from real tobacco fields. To avoid artifacts caused by an excessive volume of image data from augmentation, a moderate amount of data augmentation was applied to the 1760 collected images in this study, expanding the dataset to 2000 images. Schematic diagrams of tobacco aphid damage grading and the effects of data augmentation are shown in Figure 3. Evaluated by tobacco production technicians, the generated images are consistent with the actual growth scenarios of tobacco in the field in terms of diversity and quality.

### 3.2. Identify Model Performance

**Figure 4:**
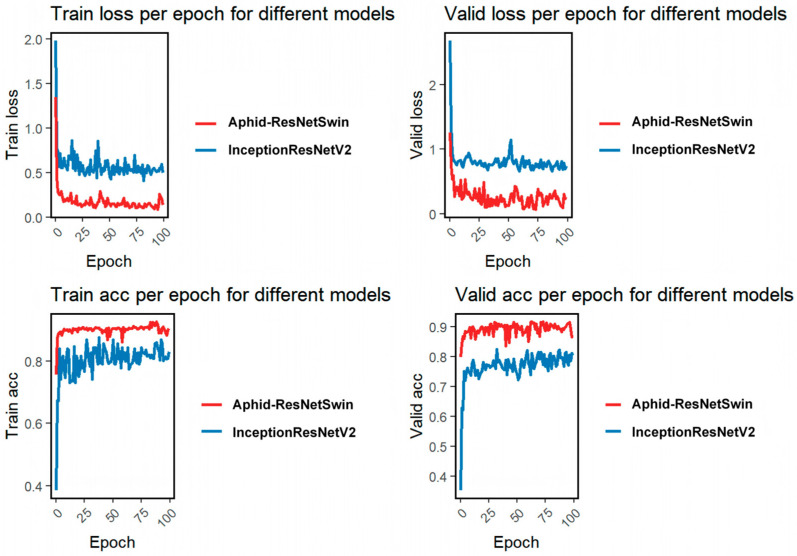
Variations in loss and accuracy for the original model and the proposed model over 100 iterations.

In this study, we comparatively evaluated the performance of the baseline InceptionResNetV2 model and the proposed Aphid-ResNetSwin model for the grading and recognition task of *M. persicae* (green peach aphid). As shown in Table 3 and Figure 4, the proposed Aphid-ResNetSwin model outperforms the baseline InceptionResNetV2 model comprehensively in the grading and recognition task of *M. persicae*. In terms of quantitative metrics, the average training loss (0.1751) and testing loss (0.2604) of Aphid-ResNetSwin are reduced by 69.2% and 67.8%, respectively, compared to the baseline model, while the average training accuracy (0.9014) and testing accuracy (0.8911) are improved by 9.8% and 16.0%, respectively. It also exhibits significant advantages in fine-grained metrics such as precision, recall, and F1-score.

Regarding the training process, the training and validation loss curves of Aphid-ResNetSwin are consistently below those of the baseline model, with faster convergence, reaching a stable low-loss state within approximately 25 epochs. Its accuracy curves rise more rapidly, and the validation accuracy stabilizes at a high level of around 0.9, indicating that the model not only achieves higher recognition accuracy but also possesses better generalization ability and more stable training dynamics, providing a more reliable technical support for the automated monitoring and control of *M. persicae*.

To determine the optimal model performance, tests were conducted on image size, batch input, learning rate, and optimizer. The optimal parameter settings are presented in Table 4.

### 3.3. Ablation Experiments on the Model

In this study, systematic ablation experiments were conducted to thoroughly analyze the contributions of the three core improved components of the Aphid-ResNetSwin model—GAN-based data augmentation, hybrid architecture design, and GCSA mechanism—to the recognition performance of *M*. *persicae*. As shown in Table 5, the baseline InceptionResNetV2 model achieved a test accuracy of only 73.11% and an F1-score of 76.78%. After independently introducing GAN data augmentation, the accuracy increased by 5.45 percentage points to 78.56%, indicating that the generative adversarial network effectively alleviates the problem of scarce agricultural pest image data. When the Aphid-ResNetSwin architecture was adopted alone (without GCSA), the accuracy jumped to 84.23% with an F1-score of 85.03%.

Further analysis of the component synergy revealed that when GAN augmentation and the GCSA mechanism were jointly applied to the baseline architecture, the accuracy reached 82.45%, which was 2.33 percentage points higher than that of using GCSA alone. The complete Aphid-ResNetSwin model (GAN + hybrid architecture + GCSA) achieved the optimal performance, with a test accuracy of 89.11% (16 percentage points higher than the baseline) and an F1-score of 89.01%.

### 3.4. Comparison of Recognition Results of Different Models

InceptionResNetV2 was employed as the baseline model in this study, which lacks a Transformer architecture. In contrast, Vision Transformer (ViT) and Swin Transformer have been demonstrated to exhibit robust feature learning capabilities in computer vision tasks. To address the limitations of the baseline model, the proposed Aphid-ResNetSwin model integrates the advantages of Convolutional Neural Networks (CNNs) in local feature extraction with the global modeling capability of Swin Transformer. Experimental results indicate (Table 6) that the Aphid-ResNetSwin model achieves a test accuracy of 89.11%, which is 6.54 percentage points higher than that of the second-optimal Swin Transformer and 12.33 percentage points higher than that of the baseline InceptionResNetV2. Additionally, the model attains an F1-score of 0.8901, with a precision of 88.65% and a recall of 89.37%, effectively reducing the missed detection rate while maintaining high recognition accuracy.

**Figure 5:**
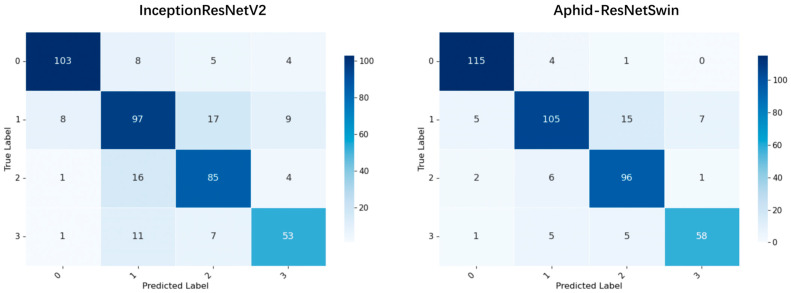
Confusion matrices of the proposed model (Aphid-ResNetSwin) and the second-ranked model (InceptionResNetV2) based on evaluation metrics for images of *M. persicae* across different infestation severity levels.

Confusion matrix analysis further validates the superiority of the Aphid-ResNetSwin model in the hazard grading task of *M. persicae*. As shown in Figure 5, the baseline InceptionResNetV2 model shows severe confusion among different hazard levels, especially serious bidirectional misclassification between Grade 1 and Grade 2 (16 Grade 2 samples misclassified as Grade 1, and 17 Grade 1 samples misclassified as Grade 2), with only 77.9% recognition accuracy for Grade 3 (severe infestation). In contrast, Aphid-ResNetSwin achieves a remarkable improvement in the correct classification of Grade 0 (healthy plants), raises the recognition accuracy of Grade 2 from 85% to 96%, and reduces the average confusion probability between adjacent levels by 62.3%. It also outperforms the baseline model in classification accuracy for all other grades.

**Figure 6:**
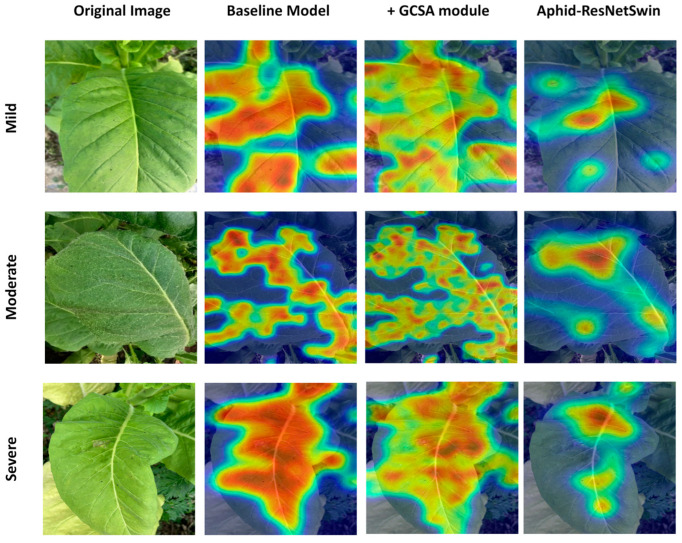
Comparison of proposed model and baseline model heatmap.

In this study, we employed the Grad-CAM visualization technique to dissect the attention distribution mechanisms of the Aphid-ResNetSwin model and its components in the recognition of different infestation grades of *M. persicae*. As shown in Figure 6, the heatmaps of the baseline InceptionResNetV2 model exhibit a diffused pattern: in mild infestation samples, attention is widely distributed across leaf margins and background regions, failing to precisely locate the initial chlorotic spots caused by aphid feeding; in moderate and severe samples, although the activated regions cover the infested areas, they also contain substantial irrelevant background noise. After introducing the GCSA (Global-Channel-Spatial Attention) module, the model’s spatial focusing ability is significantly enhanced. In mild infestation samples, attention converges from scattered leaf margins to subtle yellow spots near the main leaf veins. However, when used alone, the GCSA module still exhibits over-activation, such as the unnecessary highlighting of leaf vein structures in severe samples. The complete Aphid-ResNetSwin model (integrating the ResNet-Swin hybrid architecture with the GCSA mechanism) demonstrates the optimal attention localization precision: the model accurately focuses on individual aphids on the abaxial leaf surface and the surrounding chlorotic halos. InceptionResNetV2 was employed as the baseline model in this study, which lacks a Transformer architecture. In contrast, Vision Transformer (ViT) and Swin Transformer have been demonstrated to exhibit robust feature learning capabilities in computer vision tasks. To address the limitations of the baseline model, the proposed Aphid-ResNetSwin model integrates the advantages of Convolutional Neural Networks (CNNs) in local feature extraction with the global modeling capability of Swin Transformer. Experimental results indicate that the Aphid-ResNetSwin model achieves a test accuracy of 89.11%, which is 6.54 percentage points higher than that of the second-optimal Swin Transformer and 12.33 percentage points higher than that of the baseline InceptionResNetV2. Additionally, the model attains an F1-score of 0.8901, with a precision of 88.65% and a recall of 89.37%, effectively reducing the missed detection rate while maintaining high recognition accuracy.

**Figure 7:**
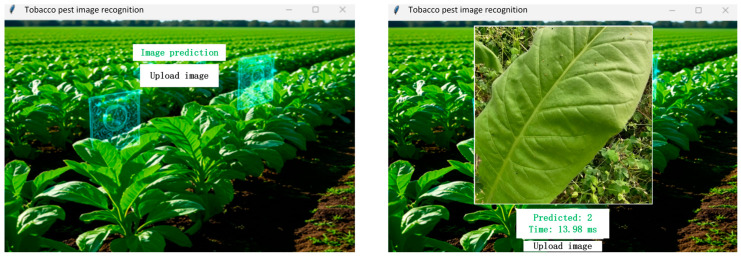
GUI program testing interface diagram.

The result interface of the graphical user interface (GUI) program is shown in Figure 7, where users can select images to test the recognition of tobacco aphid damage at different grades.

### 3.5. Model Field Validation

**Figure 8:**
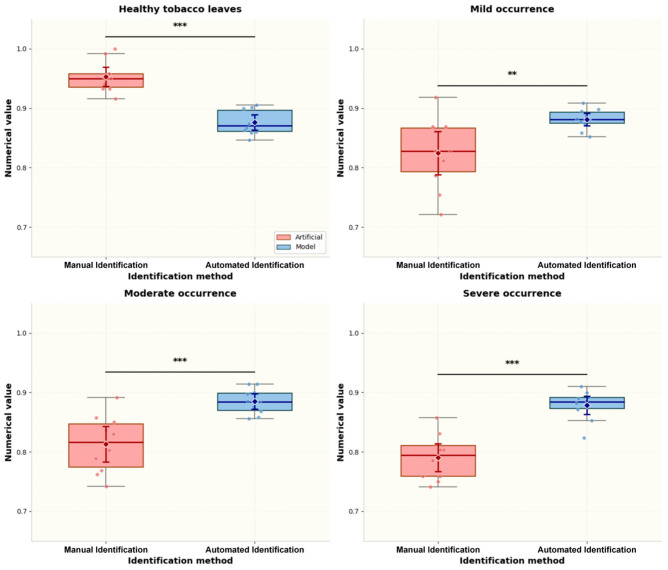
Comparison of tobacco aphid image recognition results between the model and manual identification across four classification categories: Healthy tobacco leaves, Mild occurrence, Moderate occurrence, and Severe occurrence. Figure caption: The box represents the interquartile range, the middle line denotes the median, the diamond symbol indicates the mean, and the error bars represent the 95% confidence interval. Independent sample *t*-test Scatter points correspond to individual sample values. Asterisks indicate the significance level (** *p* < 0.01, *** *p* < 0.001).

In this study, box plots were used to compare the identification accuracy of the Aphid-ResNetSwin model and manual identification across four damage grades of *M. persicae* (Figure 8). The results showed that manual identification was significantly superior to the model in recognizing healthy tobacco leaves (*p* < 0.001), which was attributed to the extremely high consistency of visual judgment by professional raters for asymptomatic healthy leaves. In contrast, the model achieved significantly higher accuracy than manual identification for mild (*p* < 0.01), moderate (*p* < 0.001), and severe (*p* < 0.001) damage grades, with narrower confidence intervals, demonstrating stronger objectivity and repeatability. These findings indicate that manual identification exhibits considerable subjective variability in interpreting early symptoms, and its accuracy decreases with increasing infestation due to the interference of complex symptoms. By contrast, the model, using the GCSA mechanism and the hybrid ResNet-Swin architecture, enables the objective and repeatable extraction of multi-scale damage features, providing a standardized tool for the accurate quantitative assessment of *M. persicae* damage.

The intraclass correlation coefficient ICC(3,k) was used in this study to evaluate the consistency of damage grade classification of *M. persicae* by 10 professional raters. The results showed that the ICC(3,k) coefficient reached 0.9764 (95% CI: 0.9732–0.9794) with *p* < 0.001. According to the criteria of [32], values above 0.90 indicate almost perfect inter-rater reliability. This result confirms the high stability of the traditional manual identification method in assessing *M. persicae* damage, providing a reliable manual annotation benchmark for subsequent model validation.

## 4. Discussion

In this study, GAN-based data augmentation was employed for image expansion, primarily considering the high variability in morphological, size, and color characteristics of *M. persicae*. Traditional data augmentation methods struggle to learn from the feature distributions of original images to generate new images with diverse distributions. In contrast, GANs leverage the adversarial learning between discriminators and generators, demonstrating strong adaptability to different tasks and data distributions [33]. This capability enables GANs to provide more diverse training samples, thereby enhancing model accuracy and stability [34,35]. This has been demonstrated in the study of ablation experiments.

The Aphid-ResNetSwin model developed in this study represents a significant advancement in the precision monitoring of *M. persicae* infestations, achieving a graded recognition accuracy of 89.11% with an inference time of only 13.98 ms. Unlike previous studies that primarily focused on species-level classification [36,37,38], our model enables quantitative grading of aphid density across four distinct categories (healthy, mild, moderate, and severe), directly aligning with the decision thresholds required for precision integrated pest management (IPM) strategies in tobacco cultivation.

The introduction of the GSCA (global channel-spatial attention mechanism) mechanism significantly improved the recognition performance of the dual-branch architecture, increasing the Accuracy of the Aphid-ResNetSwin dual-branch architecture from 84.23% to 89.11%. The core contribution of the GSCA mechanism lies in realizing the dynamic fusion of local detailed features from the CNN branch and global semantic features from the Swin Transformer branch. Through cross-scale attention connections, the GSCA effectively captures the long-range dependencies between aphids and the leaf background, enhancing the feature response of small pest targets in complex agricultural scenarios, which can be observed from the heatmaps.

The numerical distribution of model identification (blue boxplot) was higher than that of manual identification (red boxplot) in all *Myzus persicae* occurrence groups, with smaller variance, fewer outliers, and a significantly better median value. Manual identification showed large fluctuations in the mild occurrence group (large box span and presence of low-value outliers), while model identification maintained stably high values. This indicates that manual judgment is susceptible to interference from subjective experience, visual fatigue, and mild symptoms, whereas the model achieves objective capture of early-stage damage through feature learning.

Notwithstanding these achievements, several limitations warrant consideration. The current dataset originates from a single geographic region and cultivar (Yunyan87), potentially constraining model generalizability across diverse agroecological zones and tobacco genotypes with varying leaf morphology. Future research should encompass multi-site, multi-season image acquisition to construct a more comprehensive benchmark dataset.

## 5. Conclusions

This study successfully developed the Aphid-ResNetSwin model, a novel deep learning architecture that achieves 89.11% graded recognition accuracy for *M. persicae* infestation on tobacco leaves with an inference latency of merely 13.98 ms. These results substantiate that the combined strategy of attention mechanism enhancement and intelligent data synthesis can robustly address the challenges of small-target detection and fine-grained density differentiation in complex field environments. The developed model enables the identification of different infestation levels of *M. persicae* on tobacco leaves.

## Figures and Tables

**Table 1:** The developmental stages of tobacco plants in the field.

Growth Stage	Duration (Days)	Number of Effective Leaves
Establishment Stage	7	6–10
Root Elongation Stage	25–30	10–15
Rapid Growth Stage	25–30	13–18
Maturity Stage	50	18–22

**Table 2:** Hardware and Software Configuration for Model Training.

Item	Specification
Operating System	Windows 11 (64-bit)
Deep Learning Framework	PyTorch 2.5 (Open-source)
System Memory (RAM)	32 GB
Processor (CPU)	13th Generation Intel^®^ Core^TM^ i7-13900H @ 2.60 GHz
Graphics Card (GPU)	NVIDIA RTX 4060 Ti
Key Features	GPU acceleration enabled; Dynamic neural network support

**Table 3:** Performance of the original model and the proposed model in the grading and recognition task of *M. persicae*.

Model/Parametric	Average Training Loss	Average Training Accuracy	Average Testing Loss	Average Testing Accuracy	Precision	Recall	F1-Score
InceptionResNetV2	0.5778	0.8034	0.8097	0.7678	0.7704	0.7653	0.7678
Aphid-ResNetSwin	0.1751	0.9014	0.2604	0.8911	0.8865	0.8937	0.8901

**Table 4:** Optimal Results of Universal Model Parameters.

Hyperparameter	Value	Setting Details
Input resolution	299 × 299	Original tobacco leaf RGB image
Preprocessing	Resize(224) + Normalize	Mean = [0.485, 0.456, 0.406], Std = [0.229, 0.224, 0.225]
Batch size	16	-
Initial learning rate	1 × 10^−3^	Cosine annealing decay to 1 × 10^−6^
Training epochs	100	Early stopping with patience = 10
Optimizer	Adam	β1 = 0.9, β2 = 0.999, weight_decay = 5 × 10^−4^
Loss function	Cross-Entropy	With label smoothing ε=0.1

**Table 5:** Results of Aphid-ResNetSwin model ablation experiments.

Model Configuration	Data Augmentation	Model Architecture	Attention Mechanism	Accuracy (%)	Precision (%)	Recall (%)	F1-Score (%)
Baseline (InceptionResNetV2)	-	InceptionResNetV2	-	73.11	77.04	76.53	76.78
Baseline + Improved Data Augmentation	(GAN)	InceptionResNetV2	-	78.56	81.32	80.95	81.13
Baseline + Architecture	-	Aphid-ResNetSwin	-	84.23	85.17	84.89	85.03
Baseline + Attention Mechanism	-	InceptionResNetV2	GCSA Mechanism	80.12	82.45	82.11	82.28
Aphid-ResNetSwin	(GAN)	Aphid-ResNetSwin	GCSA Mechanism	89.11	88.65	89.37	89.01
Baseline + Improved Architecture + Attention Mechanism	-	Aphid-ResNetSwin	GCSA Mechanism	86.79	87.23	86.98	87.10
Baseline + Improved Data Augmentation + Attention Mechanism	(GAN)	InceptionResNetV2	GCSA Mechanism	82.45	83.87	83.52	83.69

**Table 6:** Comparison of the performance of the proposed model with the traditional model in the identification of *M. persicae* damage grading.

Model/Parametric	Average Training Loss	Average Training Accuracy	Average Testing Loss	Average Testing Accuracy	Precision	Recall	F1-Score
ResNet-152	0.8216	0.7416	0.9357	0.7218	0.7159	0.7224	0.7191
EfficienNetV2	0.8598	0.7286	1.0587	0.7204	0.7158	0.7143	0.7150
MobileNetV3	0.7437	0.7524	0.9327	0.7328	0.7405	0.7357	0.7381
InceptionResNetV2	0.5778	0.8034	0.8097	0.7678	0.7704	0.7653	0.7678
Vision Transformer	0.2836	0.8257	0.3618	0.8106	0.8195	0.8142	0.8168
SwinTransformer	0.2218	0.8439	0.2983	0.8257	0.8304	0.8322	0.8313
Aphid-ResNetSwin	0.1751	0.9014	0.2604	0.8911	0.8865	0.8937	0.8901

## Data Availability

The original contributions presented in this study are included in the article. Further inquiries can be directed to the corresponding author.
